# Feline immunodeficiency virus, feline leukemia virus and *Leishmania* spp. prevalence in cats from shelters in Mato Grosso do Sul, Brazil

**DOI:** 10.1590/S1984-29612024035

**Published:** 2024-06-28

**Authors:** Walderson Zuza Barbosa, Karen Araújo Magalhães, Kamily Fagundes Pussi, Manoel Sebastião da Costa Lima, Herintha Coeto Neitzke-Abreu

**Affiliations:** 1 Programa de Pós-graduação em Ciências da Saúde, Faculdade de Ciências da Saúde, Universidade Federal da Grande Dourados – UFGD, Dourados, MS, Brasil; 2 Faculdade de Ciências da Saúde, Universidade Federal da Grande Dourados – UFGD, Dourados, MS, Brasil; 3 Instituto Aggeu Magalhães, Fundação Oswaldo Cruz, Recife, PE, Brasil

**Keywords:** PCR, Leishmaniasis, nasal mucosa, conjunctival mucosa, FIV, FeLV, PCR, Leishmaniose, mucosa nasal, mucosa conjuntival, FIV, FeLV

## Abstract

Diseases such as those caused by feline immunodeficiency virus (FIV) and feline leukemia virus (FeLV) represent health problems for cats. Feline leishmaniasis (FL) has been reported in several cities across the country. The objective was to carry out a clinical-epidemiological and laboratory study of FIV, FeLV and FL in cats from shelters in Dourados, Mato Grosso do Sul, Brazil. Blood samples and swabs from the conjunctival and nasal mucosa were obtained from 75 cats, from four animal shelters. Serology for FIV and FeLV was performed. For *Leishmania*, polymerase chain reaction (PCR) was performed on blood, conjunctiva and nasal mucosa. In the immunochromatographic serological test, seven cats tested positive for FIV and none for FeLV. No samples was positive in PCR for *Leishmania*. The study showed that despite the presence of human and canine leishmaniasis in the studied region, *Leishmania* spp. were absent in the cats studied. To avoid an increase in contagion in shelters, it is essential isolate cats with FIV.

## Resumo

Doenças como as causadas pelos vírus da imunodeficiência felina (FIV) e vírus da leucemia felina (FeLV) representam problemas de saúde para os gatos. A leishmaniose felina (LF) tem sido relatada em diversas cidades do país. O objetivo deste trabalho foi realizar um estudo clínico-epidemiológico e laboratorial de FIV, FeLV e LF em gatos de abrigos em Dourados, Mato Grosso do Sul, Brasil. Amostras de sangue e swabs da mucosa conjuntival e da mucosa nasal foram obtidas de 75 gatos, dos quatro abrigos de animais. Foi feita a sorologia para FIV e FeLV. Para *Leishmania* foi realizada a reação em cadeia da polimerase (PCR) em sangue, conjuntiva e mucosa nasal. No teste sorológico imunocromatografico, sete gatos apresentaram resultado positivo para FIV e nenhum para FeLV. Nenhuma amostra foi positiva na PCR para *Leishmania*. O estudo demonstrou que apesar da presença de leishmaniose humana e canina, na região estudada, não foi encontrado *Leishmania* spp. nos gatos analisados. Para evitar o aumento do contágio em abrigos é fundamental isolar os gatos com FIV.

## Introduction

Common retroviral diseases, such as feline immunodeficiency virus (FIV) and feline leukemia virus (FeLV), pose health problems for cats. The most affected animals are adult males with access to the external environment (Gleich & Hartmann, 2009).

Diseases caused by hemoparasites and retroviruses are emerging challenges that affect cats in many parts of the world. Free-range cats are highly vulnerable to infections because of exposure to a variety of ectoparasites that can transmit pathogens (Otranto & Dantas-Torres, 2010; Tasker, 2010). Leishmaniasis is endemic in the state of Mato Grosso do Sul (MS) and should be further investigated. Feline leishmaniasis (FL) has been reported in several cities in the Brazil, including Campo Grande/MS (Coelho et al., 2011;Benassi et al., 2017;Metzdorf et al., 2017; Asfaram et al., 2019;Mendonça et al., 2020).

Therefore, owing to the endemic characteristics of leishmaniasis in the state and as the main causes of infectious diseases in cats, this study aimed to conduct a clinical-epidemiological and laboratory study of FIV, FeLV and FL in cats from shelters in Dourados, MS, Brazil.

## Material and Methods

### Study population

Males and females domestic cats, different ages and mixed breed were examined in all cat shelters in the city of Dourados, Brazil: 25 cats in shelter 1 (22.22680°S, 54.84430ºW) with 95 cats sheltered, 25 cats in shelter 2 (22.27064ºS, 54.75491ºW) with 68 cats, all 15 cats from shelter 3 (22.21412ºS, 54.75907ºW), and 10 cats in shelter 4 (22.20158ºS, 54.87498ºW) with 12 cats sheltered. The sample for the study was calculated based on a total population of 190 cats, considering a confidence level of 95% and a margin of error of 6%, resulting in a sample of 75 individuals. The cats belonged to shelters, according to the owners, but had free access to the street. A cat was collected that did not belong to any shelter but had free access to it and was there at the time of sample collection. The cats were selected for convenience, from less skittish animals, which showed more apparent clinical signs and were in the shelter at the time of collection.

The animals were clinically evaluated by a veterinarian through anamnesis and physical examinations, to analyze body score, mucosal color, oral and nasal cavities, and ophthalmological and dermatological alterations.

This study was approved by the Ethics Committee on the Use of Animals (CEUA) of the Universidade Federal da Grande Dourados (protocol number 29/2020).

### Obtaining biological samples

Biological sample collection was conducted from April to May 2021. The animals were catalogued in individual files and identified by name, sex, and age. After performing clinical analysis, the animals were physically restrained to collect peripheral blood, and conjunctival and nasal swab samples.

Peripheral blood (3 mL) was collected by jugular venipuncture and placed in tubes with and without EDTA to obtain whole blood and serum, respectively. The samples were kept at -20°C for subsequent serological and molecular tests. Conjunctival and nasal samples were obtained by swab collection and stored at -20ºC in microtubes for molecular diagnosis.

### Serology for FIV and FeLV

Serological analyses were performed in serum using the Alere FIV/FeLV^TM^ Test Kit (BioNote, Abbott Diagnósticos Rápidos, Belo Horizonte, Brazil), which detects FIV IgG antibodies and FeLV antigens, following the manufacturer's protocol.

### Obtaining DNA

Genomic DNA extractions from blood, conjunctiva, and nasal samples were performed with 20% sodium dodecyl sulfate, following a previously established protocol (Neitzke-Abreu et al., 2020). For each group of samples, a positive control (blood with 10^4^ *Leishmania infantum* promastigotes) and a negative control (blood without *L. infantum*) were included.

### Polymerase chain reaction (PCR)

To search for *Leishmania*, DNA from blood, conjunctiva and nasal samples were subjected to PCR using the primers LITSR (5′-CTG GAT CAT TTT CCG ATG-3′) and L5.8S (5′-TGA TAC CAC TTA TCG GCA CTT-3′) (El Tai et al., 2000), which amplify a 320 – 350-bp fragment of the ITS1 region of *Leishmania* spp. The PCR mixture (25 μL) was composed of 0.4 μM of each primer (Exxtend Biotecnologia), 1.5 mM MgCl_2_, 0.2 mM dNTP (Invitrogen), 1.5 U Taq DNA polymerase (Phoneutria), and 2 μL DNA. Amplification was performed in a thermocycler (T100 Thermal Cycler, Bio-Rad) at 95°C for 5 min, followed by 35 cycles: 95°C for 30 s, 56.1°C for 30 s, 72°C for 1 s, and finally 72°C for 10 min. The products were kept at 4°C until electrophoresis. Amplified products were subjected to 2% agarose gel electrophoresis (KASVI, Ludwig), and stained with ethidium bromide (10 mg/mL). The presence of bands was observed using a transilluminator (Loccus Biotecnologia).

All samples were subjected to a new reaction using the primers ActinF (5’-CGG AAC CGC TCA TTG CC-3’) and ActinV (5’-ACC CAC ACT GTG CCA TCT A-3’) (Du Breuil et al., 1993), which amplify a 289-bp fragment present in the mammalian β-actin gene, as a control for DNA quality. The PCR mixture (15 μL) was composed of 0.4 μM of each primer (Exxtend Biotecnologia), 1.5 mM MgCl_2_, 0.2 mM dNTP (Invitrogen), 1 U Taq DNA polymerase (Phoneutria), and 1 μL DNA. Amplification was performed in a thermocycler (T100 Thermal Cycler; Bio-Rad) at 95°C for 5 min, followed by 35 cycles: 94°C for 30 s, 57°C for 30 s, 72°C for 1 min, and finally at 72°C for 10 min. The products were kept at 4ºC until subsequent electrophoresis on agarose gel was performed.

## Results

Samples were collected from 75 domestic cats (45 males and 30 females), aged between 4 months and 11 years, mixed breed. Of the animals analyzed, 56% (42/75) exhibited some clinical signs, among which the main manifestations observed were: nasal secretion 18%, skin disease 13%, gingivitis-stomatitis complex 12%, weight loss 9.3%, pale mucous membranes 6.6%, ectoparasites 6.6%, ocular discharge 5.3%, lymphadenopathy 4%, jaundice 2.6%, blepharitis 2.6% and keratoconjunctivitis sicca 3%. In some cases, diarrhea (5.3%) was observed at the time of collection and bloody urine characterizing a urinary tract infection (5.3%).

Seven cats tested positive for FIV and no cat tested positive for FeLV. Of the FIV-positive cats, only three had clinical signs and presented oral lesions, alopecia and ear tip lesions. None of the animals tested positive for *Leishmania* spp. in the analyzed samples. The internal control (PCR actinF/V) showing the presence of mammalian DNA in all samples tested.

## Discussion

Cats are common domestic animals in homes, and approximately 27.1 million felines in Brazil are estimated to share the same environment as humans (Instituto Pet Brasil, 2022). It is important to understand and investigate feline pathogens that may be involved in human diseases, owing to this close contact, which can facilitate the sharing of pathogens (Brum et al., 2007). In addition to the overcrowding of animals, shelter environments are usually precarious and favorable for FL and other diseases, because of the accumulation of organic matter (environments with fruit trees and shady places), a worrying fact because leishmaniasis vectors lay eggs in these places (Brasil, 2014).

Owing to the role of dog as a primary reservoir and the severe disease that can occur, leishmaniasis in dogs has been well studied. In contrast, cats have been considered, for many years, as less susceptible or resistant animals, and their role in the epidemiology of leishmaniasis is considered insignificant. Despite the initial reluctance, cases of FL have been increasingly reported worldwide.

Most cats with FL are subclinically infected, possibly because of their natural resistance to the development of disease (Solano-Gallego et al., 2007). The most common clinical signs include lymphadenomegaly, cutaneous and mucocutaneous lesions (nodular and/or ulcerative dermatitis), with or without visceral signs (Pennisi, 2015), generalized weakness, weight loss, anorexia, and ocular and oral lesions (Pennisi & Persichetti, 2018). However, not all clinical signs in cats with FL are necessarily due to this disease; some may be caused by comorbidities, such as FIV and FeLV (Marcos et al., 2009). Retroviruses are associated with various clinical problems such as anemia, lymphoma, chronic inflammatory disease, oral inflammatory disease such as gingivostomatitis, and secondary and opportunistic infections (Little et al., 2020).

In the present study, three cats showed clinical signs of FIV (oral lesions, alopecia, and ear tip lesions), and one of them did not live in the shelter but had free access to and contact with the resident cats. This wandering animal, which has free access to the shelter, becomes a source of infection for other cats, demonstrating a health risk. Although none of the cats examined tested positive for *Leishmania* spp., studies have demonstrated the presence of *Leishmania* in cats. A study carried out in Israel showed that 75% (50/67) of cats housed in a shelter were seropositive for *L. infantum*, and 16% (11/67) were positive in the DNA analysis using PCR (Baneth et al., 2020). Peripheral blood PCR was negative for *Leishmania* spp., a very low result compared to the rates observed in another region of MS (Metzdorf et al., 2017). Little information is available on the mechanisms responsible for the susceptibility or resistance of felines naturally exposed to infection by *L. infantum* (Persichetti et al., 2017). The PCR positivity of peripheral blood samples is generally lower than that of lymph node and bone marrow aspirates or local skin impressions (Alcover et al., 2021); however, they are less invasive. None of the shelters studied received a complaint from the city’s Zoonosis Control Center regarding precarious conditions, such as accumulation of organic matter and waste. Therefore, although the shelters have agglomerations of animals, there may be sandfly vectors in these locations.

Because retroviral diseases weaken animals and are immunosuppressive diseases, they represent a challenge for the health of felines. However, most cats naturally infected with FIV do not present a serious clinical condition; with proper care, infected cats can live for many years. As observed in the current study, although some animals tested positive for FIV, they did not present characteristic symptoms of the disease. Care must be taken in places with a high density of animals as immunosuppressive diseases favor the development of opportunistic diseases.

## Conclusion

*Leishmania* spp. were not detected in the examined cats. A negative PCR ITS1 for *Leishmania*, in blood and conjunctival and nasal samples, does not exclude *Leishmania* infection, and it is important serology, kDNA PCR and PCR of other tissues. Further investigations of FL are necessary owing to the high number of cases of human and canine leishmaniasis in Dourados, in addition to reports of the presence of sand fly vectors. Cats from shelters can have viral diseases, such as FIV and FeLV, and rapid diagnosis and early isolation of felines is important to prevent contagion to other animals. The presence of immunosuppressive diseases in cats from shelters with high animal density requires greater monitoring of the animals' health.
